# 3D Reconstruction of VZV Infected Cell Nuclei and PML Nuclear Cages by Serial Section Array Scanning Electron Microscopy and Electron Tomography

**DOI:** 10.1371/journal.ppat.1002740

**Published:** 2012-06-07

**Authors:** Mike Reichelt, Lydia Joubert, John Perrino, Ai Leen Koh, Ibanri Phanwar, Ann M. Arvin

**Affiliations:** 1 Departments of Pediatrics and Microbiology & Immunology, Stanford University School of Medicine, Stanford, California, United States of America; 2 Cell Sciences Imaging Facility, Stanford University School of Medicine, Stanford, California, United States of America; 3 Stanford Nanocharacterization Laboratory, Geballe Laboratory for Advanced Materials, Stanford University, Stanford, California, United States of America; University of Glasgow, United Kingdom

## Abstract

Varicella-zoster virus (VZV) is a human alphaherpesvirus that causes varicella (chickenpox) and herpes zoster (shingles). Like all herpesviruses, the VZV DNA genome is replicated in the nucleus and packaged into nucleocapsids that must egress across the nuclear membrane for incorporation into virus particles in the cytoplasm. Our recent work showed that VZV nucleocapsids are sequestered in nuclear cages formed from promyelocytic leukemia protein (PML) *in vitro* and in human dorsal root ganglia and skin xenografts *in vivo*. We sought a method to determine the three-dimensional (3D) distribution of nucleocapsids in the nuclei of herpesvirus-infected cells as well as the 3D shape, volume and ultrastructure of these unique PML subnuclear domains. Here we report the development of a novel 3D imaging and reconstruction strategy that we term Serial Section Array-Scanning Electron Microscopy (SSA-SEM) and its application to the analysis of VZV-infected cells and these nuclear PML cages. We show that SSA-SEM permits large volume imaging and 3D reconstruction at a resolution sufficient to localize, count and distinguish different types of VZV nucleocapsids and to visualize complete PML cages. This method allowed a quantitative determination of how many nucleocapsids can be sequestered within individual PML cages (sequestration capacity), what proportion of nucleocapsids are entrapped in single nuclei (sequestration efficiency) and revealed the ultrastructural detail of the PML cages. More than 98% of all nucleocapsids in reconstructed nuclear volumes were contained in PML cages and single PML cages sequestered up to 2,780 nucleocapsids, which were shown by electron tomography to be embedded and cross-linked by an filamentous electron-dense meshwork within these unique subnuclear domains. This SSA-SEM analysis extends our recent characterization of PML cages and provides a proof of concept for this new strategy to investigate events during virion assembly at the single cell level.

## Author Summary

Varicella-zoster virus (VZV), the cause of varicella and zoster, is a human herpesvirus that replicates in the host cell nucleus where viral genomes are packaged into virion nucleocapsids. We have recently identified antiviral PML (promyelocytic leukemia) nuclear cages that sequester VZV nucleocapsids and inhibit formation of infectious particles. Here we developed a novel three-dimensional (3D) imaging and reconstruction strategy, termed Serial Section Array-Scanning Electron Microscopy (SSA-SEM) that together with electron tomography made it possible to derive 3D reconstructions of complete herpesvirus infected host cell nuclei and of PML cages with ultrastructural precision for the first time. We determined the 3D distribution of several thousand nucleocapsids within reconstructed volumes of single host cell nuclei and in PML cages as well as their sequestration efficiency and sequestration capacity: more than 98% of nucleocapsids were entrapped within PML cages and individual PML cages could sequester nearly 3,000 nucleocapsids which were cross-linked by an irregular electron-dense meshwork within the PML cages. This 3D analysis provides a proof of concept for using SSA-SEM to investigate virion assembly at the whole cell level and further elucidates our observation that PML cages are antiviral nuclear domains which block VZV nucleocapsid egress from the infected cell nucleus.

## Introduction

Varicella-zoster virus (VZV) is an alphaherpesvirus that causes varicella (chickenpox) and herpes zoster (shingles) [1]. The host range of VZV is restricted to humans and its life cycle in the human host depends upon tropism for skin, lymphocytes and neurons in sensory ganglia, where it establishes latency [1], [2]. VZV pathogenesis can be investigated *in vivo* using xenografts of human dorsal root ganglia (DRG) and skin in a severe combined immunodeficiency (SCID) mouse model [3], [4]. Since VZV infectious particles are highly cell-associated, VZV spreads from cell to cell, accompanied by extensive cell-cell fusion and syncytia formation *in vitro* and polykaryocyte formation in DRG and skin *in vivo* [5]–[7].

All herpesviruses, and many other DNA viruses like adenoviruses, papillomaviruses or polyomaviruses, replicate in the host cell nucleus. During VZV infection, genome copies are synthesized in nuclear replication compartments and genomic DNA is packaged into icosahedral nucleocapsids formed by ORF40, the major capsid protein, and smaller capsid surface proteins, such as ORF23 protein. After assembly, nucleocapsids egress across the nuclear membrane for secondary envelopment in the cytoplasm and are then released as enveloped infectious virus particles [1], [8].

PML protein has many different isoforms and is a major organizing component of these nuclear domains, which vary in shape, size, and molecular composition. PML isoforms share a conserved N-terminus, which is involved in PML oligomerization and contains a characteristic RBCC/TRIM motif. Different PML isoforms have unique C-terminal domains, which may be important in isoform-dependent functions [9], [10].

PML-NBs have been implicated in controlling the replication of several alphaherpesviruses [11]–[21]. PML-NBs are targeted for disassembly in VZV-infected cells in vitro and in human epidermal cells of skin xenografts infected *in vivo* by a mechanism involving the interaction of SUMO-interacting domains (SIM) of the VZV immediate early protein ORF61 with sumoylated PML protein [21]. The targeting of PML-NBs for disassembly promotes VZV replication and spread *in vivo* in human skin xenografts and depletion of PML protein enhances VZV replication in cell culture, indicating a role for PML in the host cell defense [16], [21]. Whereas PML protein undergoes little degradation in VZV-infected cells, other alphaherpesviruses, including HSV-1, pseudorabies virus (PRV), bovine herpes virus type 1 (BHV-1) and equine herpesvirus type 1 (EHV-1) target PML for immediate proteosome-mediated degradation through functions of viral ICP0 ubiquitin ligase-like proteins, albeit with different degrees of efficiency [18]. HSV-1 appears to be most strongly regulated by PML isoforms I and II, based on their capacity to partially reverse the increase in plaque formation of an ICP0-null mutant observed in PML-depleted cells [17]. Recent work showed that HSV-1 ICP0 preferentially targets SUMO-modified isoforms of PML but also triggers PML I degradation independently of SUMO modification [19]. Interestingly, PML degradation also appears to be promoted by the US3 serine/threonine kinases of HSV-2 and PRV [20].

In the case of VZV, we found that if PML nuclear bodies are not dissociated effectively, these structures function to sequester VZV nucleocapsids in differentiated human cells within DRG and skin xenografts *in vivo* and in cultured cells [22]. Large ring-like PML-NBs created cages that contained nucleocapsids sequestered in the nuclei of neurons and satellite cells [22]. These PML cages in virus-infected cells resembled PML clastosomes, which sequester aberrant polyglutamine (polyQ) proteins, such as Huntingtin (Htt), in several neurodegenerative disorders [23], [24]. Thus, entrapment of VZV nuclecapsids may reflect a more basic cytoprotective function of PML in sensing and containing nuclear aggregates of aberrant proteins in a ‘nuclear safe house’, similar to the function of nuclear aggresomes [25], [26]. Further work demonstrated that of several PML isoforms tested, only PML IV promoted the sequestration of VZV nucleocapsids through an interaction with the ORF23 capsid surface protein, and that this process significantly inhibited VZV replication *in vitro* [22].

Quantitative immuno-electron microscopy analysis of ultrathin sections indicated that the majority (about 95%) of VZV nucleocapsids were found in PML cages, suggesting a surprisingly high efficiency of PML mediated capsid sequestration [22]. However, since ultrathin sections cannot reveal the shape and volume of PML cages, it was not possible to determine their sequestration capacity, that is, how many VZV nucleocapsids may be sequestered inside individual PML cages. Furthermore, because ultrathin (50–100 nm) cross-sections through a nucleus may represent <1% of the diameter of a typical mammalian cell nucleus the sequestration efficiency, defined as the proportion of all nucleocapsids present in a complete individual nucleus that are sequestered within PML cages, could not be determined.

The goal of this study was to develop an EM imaging method with a high enough resolution to precisely identify, locate and count VZV nucleocapsids and at the same time, allow the efficient 3D reconstruction of large volumes of host cell nuclei, including complete PML cages. Here we describe a novel 3D imaging and reconstruction strategy that we term Serial Section Array-Scanning Electron Microscopy (SSA-SEM). Using this method together with electron tomography, we were able to create 3D reconstructions of complete nuclei of herpesvirus-infected cells and of PML cages with sequestered VZV nucleocapsids. Determining the shape and the volume of host cell nuclei and PML cages together with the precise 3D localization of several thousand VZV nucleocapsids enabled us for the first time to quantitatively estimate the sequestration capacity and efficiency of individual PML nuclear cages. The application of this strategy to resolve questions about PML-NB entrapment of VZV nucleocapsids is a proof of concept for its use to address other questions in virology and cell biology.

## Results

### PML cages with sequestered VZV nucleocapsids can be visualized by scanning EM

**Figure 1:**
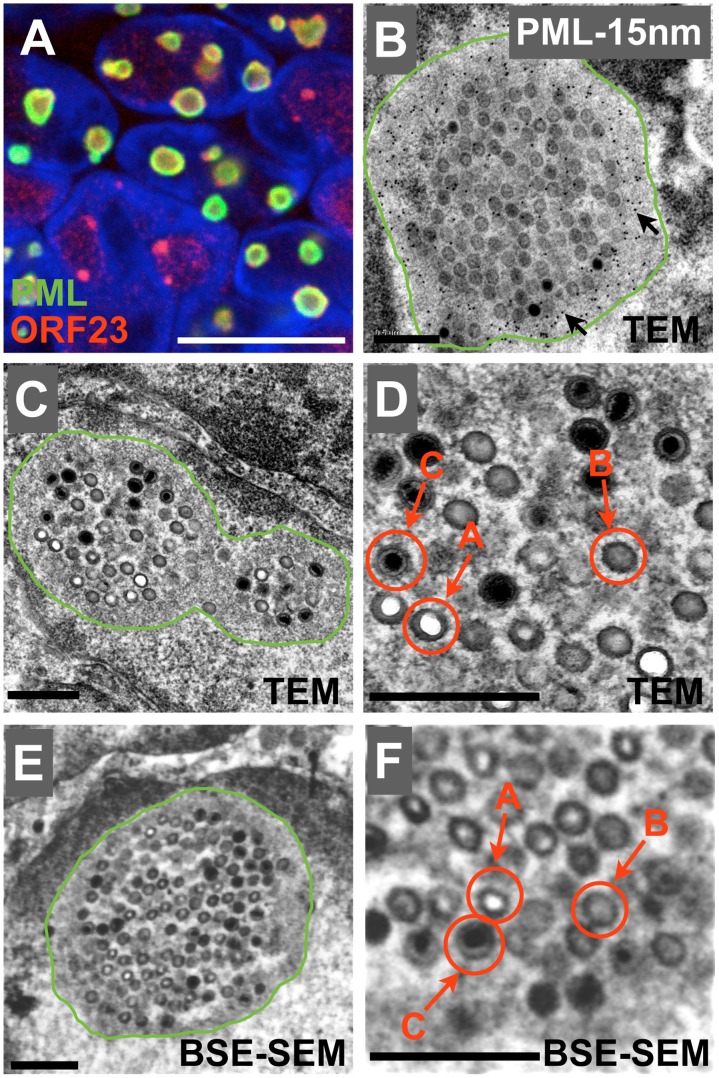
Visualization of PML cages and VZV nucleocapsids by confocal microscopy, TEM and SEM. (A–F) Melanoma cells that expressed doxycycline-induced PML IV were infected with VZV for 48 hours. (A) Analysis by confocal microscopy: permeabilized cells on coverslips were immunostained for PML (green) and ORF23 capsid protein (red); nuclei were stained with Hoechst (blue). Scale bar, 10 µm. (B) Immunogold TEM analysis: cells were high-pressure frozen, freeze-substituted, embedded in LR-White resin and then labeled with anti-PML polyclonal rabbit antibody and Protein A conjugated with 15 nm gold particles. Note the dense PML-gold labeling (arrows) in the amorphous layer (surrounded by a green line) that encloses the sequestered capsids. (C and D). Standard TEM for morphological analysis: cells were aldehyde-fixed, ‘en block’ stained for high contrast and then embedded in epoxy-resin. Note the electron dense amorphous PML layer (surrounded by green line) that encloses the clustered capsids. (D) Three types of capsids (A, B, C-type capsids, red arrows) can be distinguished by TEM. (E and F) Scanning EM analysis with a back-scattered electron detector (BSE) of the same sample as in C. Note the electron dense PML layer (surrounded by a green line) that encloses the sequestered capsids. (F) The three types of capsids (A, B, C-type capsids, red arrows) can also be distinguished by BSE-SEM. Scale bars in B–F are 500 nm.

As we have shown previously [22], PML cages in VZV-infected cells appear as ring-like structures that contain ORF23 capsid protein by confocal microscopy using antibodies to PML and ORF23, the small capsid protein (Figure 1A). At the higher resolution obtained by immunogold-TEM, mature (C-type capsids) and immature (A-and B-type capsids) can be identified that are embedded within and surrounded by densely immunogold-labeled PML positive material (Figure 1B). Next, we employed a high-contrast sample preparation protocol in order to be able to identify PML cages solely by their distinct morphology in samples not suitable for immunogold labeling. Similar to the densely labeled PML shell visible by immunoTEM (Figure 1B), a shell of amorphous electron dense material surrounding clusters of VZV nucleocapsids was visible in the high-contrast embedded samples (Figure 1C, green line). Sequestered mature C-type capsids and immature A-and B-type capsids could be distinguished clearly (Figure 1D). Importantly, the electron dense PML-positive shell surrounding sequestered VZV nucleocapsids was also visualized when the same sample was studied using a high-resolution scanning electron microscope (SEM) equipped with a field emission gun (FEG) and a back-scattered electron detector (BSE-detector) (Figure 1E) and the SEM resolution was sufficient to distinguish between mature and immature nucleocapsids (Figure 1F). Therefore, the distinctive morphological profiles of nuclear PML cages that contain sequestered nucleocapsids could be identified unequivocally by TEM as well as SEM. These results made it possible to perform the large volume and high-resolution 3D reconstruction of VZV-infected cell nuclei and PML cages aided by SEM imaging.

### Serial section array-scanning electron microscopy (SSA-SEM)

VZV-infected cell nuclei with diameters of about 5–10 µm and PML cages of 0.5-5 µm diameter [22] are too large to be fully reconstructed by conventional electron tomography approaches that usually use 100–300 nm sections. Although a serial ultrathin section approach in combination with TEM analysis could be used to reconstruct whole nuclei or cells, this approach has proven very time consuming and has several technical disadvantages. Since the area on one TEM grid is very small, several grids must used if imaging more than 10–20 serial sections is necessary to create a large volume reconstruction; TEM grids are easily damaged and damage to just one grid means that the whole series of sections before and after the missing grid cannot be used for the 3D reconstruction experiment. TEM sections are also prone to folding when placed on the grid, which causes distortions in the 3D reconstruction. In contrast, large samples fit into the microscope for SEM and long ribbons of ultrathin sections can be deposited on glass slides.

**Figure 2:**
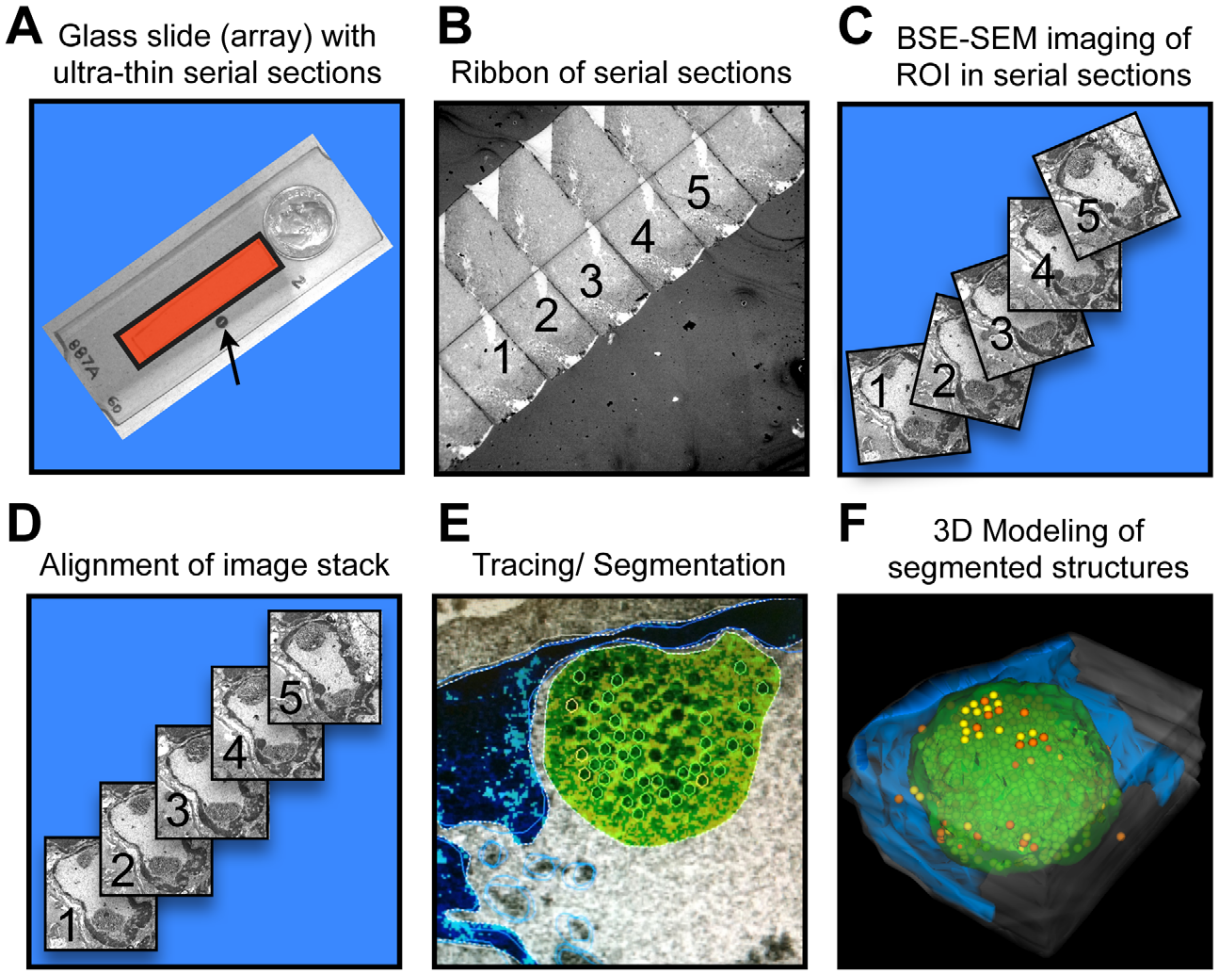
Outline of the serial section array scanning electron microscopy (SSA-SEM) method. SSA-SEM enables the three-dimensional reconstruction of cell nuclei and PML domains combined with the visualization and quantification of VZV capsids with ultrastructural precision. (A) Ribbons of ultrathin serial sections are placed on gelatin-coated glass slides and then carbon-coated to prevent charging effects during SEM imaging. The indicated area (red square) contains about 60 consecutive sections. A standard TEM slot-grid (arrow) commonly used in serial section TEM and a ten-cent coin are shown for size comparison. (B) Low magnification view of a ribbon of serial sections imaged by SEM using a back-scattered electron detector (BSE). (C) Using BSE-SEM, regions of interest (ROI), such as whole cells, nuclei or PML-domains can be identified and then repeatedly imaged in consecutive sections, yielding a stack of unaligned digital images of the ROI. (D) The stack of digital images must be aligned, either manually or automatically, for later 3D reconstruction. (E) Structures of interest, such as electron dense heterochromatin (blue), PML domains (green) and VZV capsids (yellow) are manually or automatically (threshold) traced in each serial section for quantification of numbers, areas or volumes and for the visualization of size, shape and distribution of segmented structures in the final 3D model (F).

We developed SSA-SEM as a method that provided both a high enough resolution to identify and precisely locate virion capsids and at the same time allowed the efficient 3D reconstruction of large volumes of host cell nuclei and complete PML cages (Figure 2). SSA-SEM combines principles and strategies of related methods such as immunofluorescence (IF) array tomography [27], [28], serial block face-SEM [29] and focus ion beam (FIB) or iron abrasion SEM [30], [31]. Ribbons of ultrathin serial sections were acquired by ultramicrotomy. We used 100 nm sections to avoid double counting of VZV nucleocapsids, which have a diameter of approximately 100 nm, in consecutive sections. Ribbons of serial sections were transferred onto gelatin-coated glass-slides (Figure 2A), followed by heavy metal counterstaining and a final carbon coating step to avoid charging during SEM imaging. The serial section array was then imaged with a high-resolution SEM using a BSE detector, which generates TEM-like images of cell structures with a contrast dependent mainly on the high atomic weight and differential adsorption of heavy metal stains to cellular proteins, membranes and nucleic acids (Figure 2B). Consecutive SEM imaging of serial sections created ordered stacks of unaligned digital images (Figure 2C). These stacks were then computationally aligned (Figure 2D). The aligned images were then segmented by manual or automatic (threshold) tracing of the morphology of structures of interest, e.g. nucleocapsids and PML cages (Figure 2E). From this data, a 3D model was generated that shows the shape of PML cages and the distribution of virion capsids within the reconstructed nuclear volume (Figure 2F).

### Large volume 3D reconstruction of VZV-infected cell nuclei by SSA-SEM

**Figure 3:**
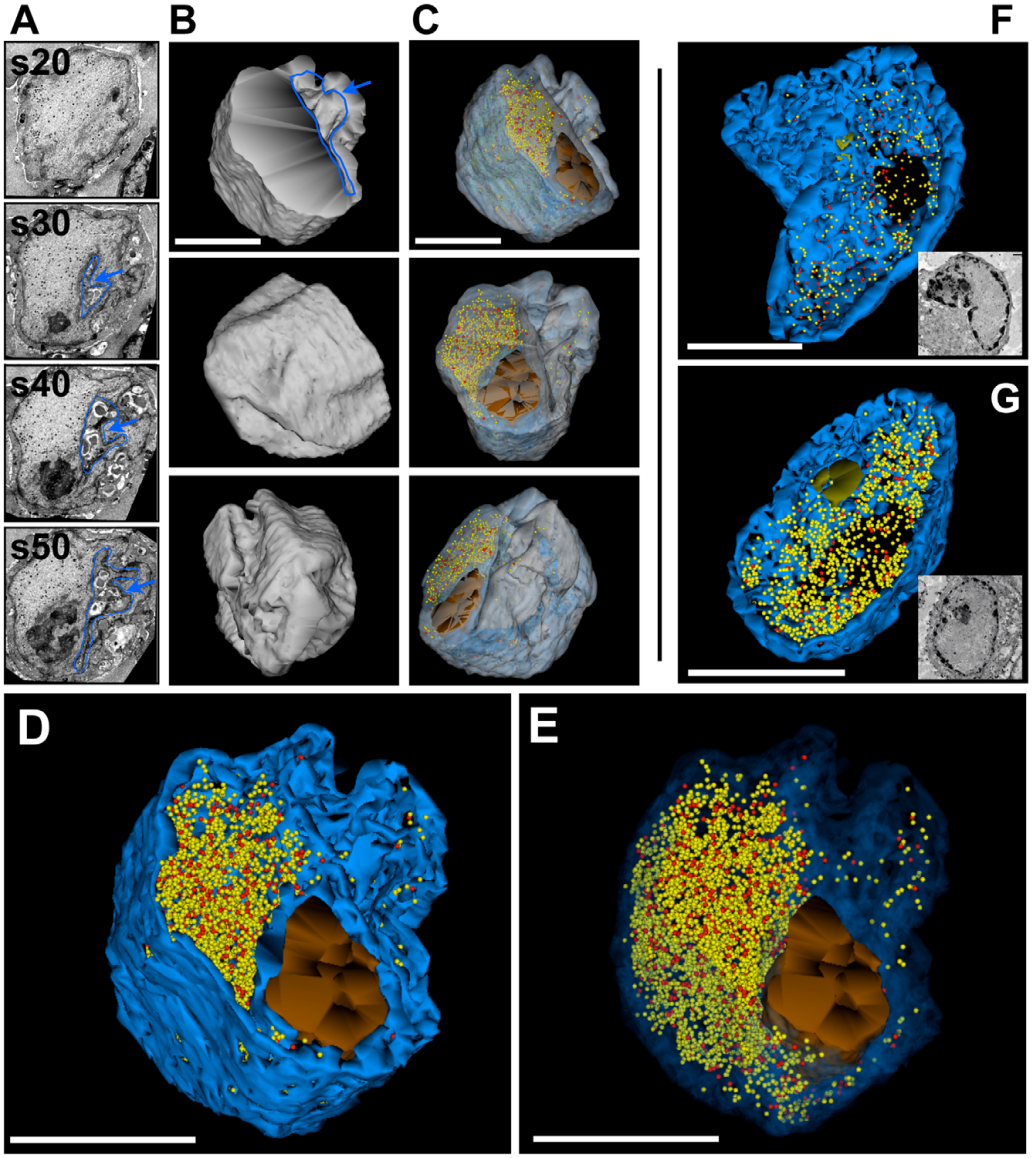
Three-dimensional distribution of VZV nucleocapsids in cell nuclei without PML cages. Melanoma cells were infected with VZV for 48 h and processed for SSA-SEM (A–E) or serial section TEM (F and G). A) BSE-SEM images of four representative sections (s20, s30, s40, s50) from a series of 50 consecutive 100 nm sections are shown. A nuclear indentation is outlined and marked with a blue arrow. See also Video S1. (B) 3D model of the shape of the VZV infected nucleus (grey). Upper panel (front view): the cross section plane and a deep invagination (blue arrow) of the nucleus are visible. The middle panel (side view) and bottom panel (rear view) reveal the irregular shape of the nucleus with numerous indentations. (C) View of the same nucleus at different angles in transparent mode. Color code: transparent grey, boundary of the nucleus; transparent blue, electron dense heterochromatin; brown, nucleolus; red spheres (mature capsids, C-type) and yellow spheres (immature capsids, A and B-type). A total of 4,223 capsids were identified and visualized. (D) Higher magnification view; color code as in C, but nuclear envelope not shown. The dense heterochromatin (solid dark blue) hides nucleocapsids that are located deeper in the nuclear volume. (E) Same view as in D, but with transparent heterochromatin: the distribution of capsids throughout the nucleus is revealed. See also Video S2. (F and G) Two different nuclei that were reconstructed from serial sections imaged by TEM. The color code is the same as above. Insets show representative images from the TEM series. The 3D models show the distribution of 425 (F) and 1,340 capsids (G), respectively. See also Video S3. All scale bars are 5 µm.

Using SSA-SEM we first analyzed a VZV-infected melanoma cell nucleus in which endogenous PML was expressed (Figure 3A–E and Video S1). The shape of the infected cell nucleus and the nuclear volume were determined by tracing the outer boundary of the nucleus in all 50 consecutive sections, encompassing a total thickness of about five microns and a nuclear volume of about 95 µm^3^. The 3D reconstruction revealed an irregular shape of the nucleus characterized by several indentations and deep invaginations (Figure 3B and Video S2). If visualization was limited to the original two-dimensional sections, these invaginations might be misinterpreted as ‘vesicles’ or ‘vacuoles’ within the nuclear matrix (Figure 3A and Video S1). Morphological tracing and 3D modeling revealed the location and distribution of the electron dense heterochromatin, which is located primarily at the periphery of the nucleus (Figure 3C–E, blue); also seen is the nucleolus in the lower center of the nucleus (Figure 3C–E, brown) and the mature and immature nucleocapsids (Figure 3C–E, red and yellow spheres, respectively). 3,467 (82%) immature capsids and 756 (18%) mature capsids were identified within the serial sections and their positions were precisely modeled in the reconstructed nuclear volume (Figure 3C–E and Video S2). This work revealed that mature and immature capsids were not segregated into different nuclear domains; instead, they were mixed randomly and were evenly distributed within the nuclear volume outside of the heterochromatin and the nucleolus and were excluded from the deep nuclear imaginations (Figure 3E and Video S2).

These SSA-SEM results were confirmed by two more 3D models of large volumes of VZV-infected cell nuclei, which were derived by morphological segmentation of 18 consecutive TEM sections of 100 nm thickness (Figures 3F and 3G; Video S3). In the reconstructed nuclear volume in Figure 3F, which accounted for 46.2 µm^3^, 109 (25.65%) mature and 316 (74.4%) immature nucleocapsids were identified and 102 (7.6%) mature and 1,238 (92.4%) immature capsids were identified in the nuclear volume in Figure 3G (43.4 µm^3^) (Figure 3G and Video S3). Similar to the nucleus in Figure 3A–E, most nucleocapsids were distributed evenly throughout the reconstructed nuclear volume; no extended clusters of aggregated nucleocapsids were visible. The quantifications of structures shown in Figure 3 are summarized in Table 1.

**Table 1:** Quantification of structures identified in 3D reconstructions of VZV infected host cell nuclei.

Figure/Object	Volume ( µm^3^)	Number of all capsids	Number of free capsids	Number of sequestered capsids	Capsid density (capsids/µm^3^)
Figure 3A–E/Nucleus 1	95	4,223 (100%)	4,223 (100%)	0	44.4
Figure 3F/Nucleus 2	46.2	425 (100%0	425 (100%)	0	9.2
Figure 3G/Nucleus 3	43.4	1,340 (100%)	1,340 (100%)	0	30.9
Figure 4B–D/Nucleus 4	63	3,062 (100%)	6 (0.2%)	3,056 (99.8%)	48.6
Figure 4B–D/Cage 1 (top)	6.2			1,732	279.4
Figure 4B–D/Cage 2 (bottom)	4.6			1,324	287.8
Figure 5A–G/Nucleus 5	291	5,597 (100%)	70 (1.3%)	5,527 (98.7%)	19.2
Figure 5A–G/Cage 1	0.8			126	157.5
Figure 5A–G/Cage 2	10			2,780	278
Figure 5A–G/Cage 3	5.8			1,778	306.5
Figure 5A–G/Cage 4	3.3			843	255.5
Average capsid packing density of PML cages 1–4 of nucleus 5 (capsids/µm^3^) ± SD					249±64 (N = 4)
Average capsid packing density of all reconstructed PML cages (capsids/µm^3^) ± SD					261±53 (N = 6)
Average capsid density in all reconstructed nuclear volumes (capsids/µm^3^) ± SD					30±17 (N = 5)

The number of free or sequestered nucleocapsids identified in VZV infected host cell nuclei and the volume of the analyzed nuclei and PML cages were determined by counting the corresponding traces in all serial sections that were used to generate the 3D models. Capsid densities were calculated by dividing the number of nucleocapsids with the corresponding volume (µm^3^) of the PML cage or nucleus. The left column indicates the corresponding figures where the quantified objects are visualized.

### Individual PML nuclear cages sequester thousands of VZV nucleocapsids

**Figure 4:**
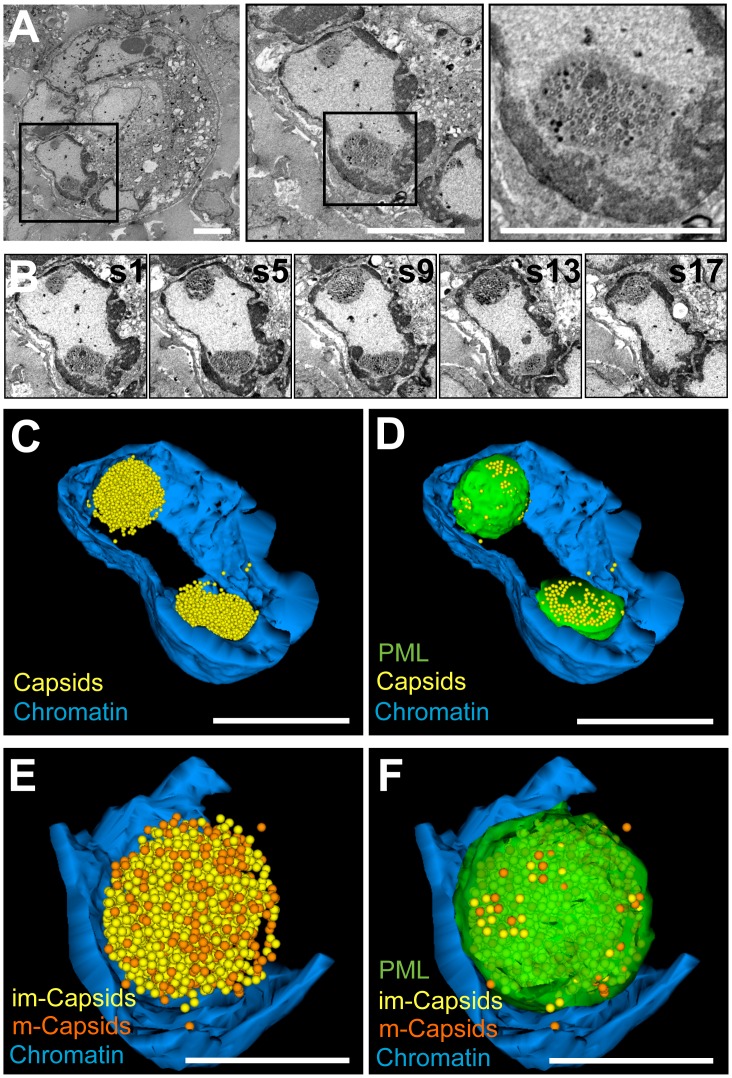
Three-dimensional distribution of VZV nucleocapsids in host cell nuclei with PML cages. Melanoma cells that express doxycycline-induced PML IV were infected with VZV for 48 h and processed for BSE-SEM imaging. (A) BSE-SEM images at different magnifications of a syncytium of VZV infected melanoma cells. Left panel: low magnification view of a syncytium; middle panel: one nucleus of the same syncytium with two PML cages; right panel: higher magnification view of a PML cage with sequestered VZV capsids. Black squares indicate areas that are shown at higher magnification in the panels to the right. Scale bars are 5 µm. (B) Five representative images (s1, s5, s9, s13, s17) from a series of 18 consecutive sections through the nucleus shown in A, middle panel. See also Video S4. (C and D) 3D models based on tracing and segmentation in all 18 sections of electron dense heterochromatin (blue); nucleocapsids (yellow spheres) and PML cages (green, shown only in D). 1,732 and 1,324 capsids were identified in the upper and lower PML cage, respectively). Scale bars are 5 µm. See also Video S5. (E and F) 3D models of the upper PML cage. Color code as above, but immature capsids (A and B-type capsids) are shown as yellow spheres and mature capsids (C-type) in orange; the PML cage is transparent green (shown only in F). Scale bars are 2 µm.

We next used SSA-SEM to analyze VZV-infected melanoma cells that express PML IV when induced with doxycycline, together with endogenous PML [22]. Inducing PML IV creates conditions that allow enough PML cages to persist in VZV-infected cells for 3D ultrastructural analysis. Using SSA-SEM we first identified a typical VZV syncytium in which infected cells are fused into a polykaryon (Figure 4A, left panel). A nucleus that contained two distinct electron dense PML cages with numerous sequestered VZV nucleocapsids was identified within the syncytium (Figure 4A, middle and right panel). Next, 18 consecutive 100 nm serial sections through this nucleus were imaged by SSA-SEM and then traced and segmented as illustrated in Figure 2. Inspecting different sections in the series (Figure 4B and Video S4) suggested a spherical shape of the PML cages. Tracing and 3D modeling (Figure 4C–F and Video S5) of the electron dense heterochromatin (blue), all nucleocapsids (yellow spheres) and mature capsids (only in Figure 4E and F, orange), and the outer surface of the electron dense shell of the PML cages (green) was performed with all 18 sections (stack thickness approximately 1.8 microns). The 3D model revealed that only six (0.2%) of a total of 3,062 nucleocapsids located within the reconstructed nuclear volume (63 µm^3^) were not aggregated together with the other nucleocapsids (yellow spheres) (Figure 4C and D). 3,056 nucleocapsids (99.8%) were in clusters enclosed by the two PML cages (green) present in this nuclear volume. Interestingly, each of the PML cages, whose reconstructed volumes were about 6.2 µm^3^ (upper cage, Figure 4D) and 4.6 µm^3^ (bottom cage in Figure 4D) contained more than a thousand nucleocapsids: 1,732 and 1,324, respectively. This information made it possible to estimate the packing density of capsids within the two PML cages, the mean of which was 284 nucleocapsids/µm^3^. A 3D model of the upper PML cage at higher magnification identified the position of both mature and immature capsids and revealed that both types were randomly packed within the PML cage (Figure 4E–F). Of note, both PML cages were associated with electron dense heterochromatin (blue) in the periphery of the nucleus (Figure 4D).

**Figure 5:**
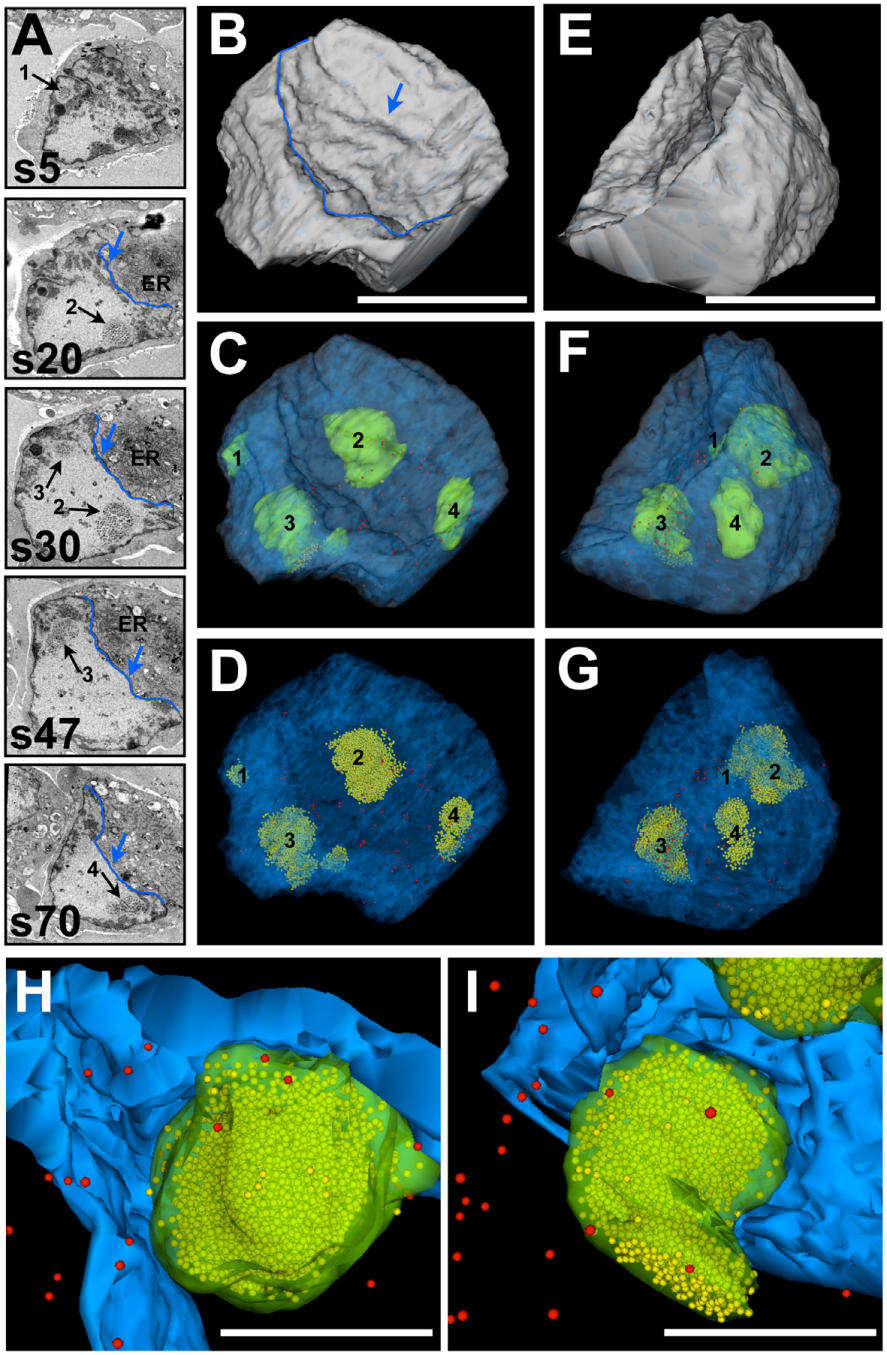
Large volume-reconstruction of a VZV infected cell nucleus with four PML cages. Melanoma cells that express doxycycline-induced PML IV were infected with VZV for 48 h and processed for BSE-SEM imaging. (A) Five representative BSE-SEM images (s5, s20, s30, s47, s70) from a series of 82 consecutive sections through a VZV nucleus with four PML cages (1–4, black arrows) with sequestered VZV capsids. A valley-like indentation of the nucleus (blue outline and arrow) and the endoplasmic reticulum (ER) are marked. See also Video S6. (B–D and E–G, respectively) show 3D models of the nucleus in two angles (100 degrees rotation to the left). (B and E) Shape of the nucleus based on tracing its outer boundary (grey). (C and F) The shape of the nucleus (transparent grey) is overlaid with the dense heterochromatin (transparent blue). PML cages 1–4 (solid green) and VZV capsids that escaped sequestration (red spheres) are visible in the interior of the nucleus. (D and G) Same view as above, but the nuclear envelope and the PML domains are completely transparent. This reveals the location of all VZV capsids (5,597) identified in this nucleus; red spheres represent free capsids (70) and yellow spheres represent sequestered capsids (5,527). Scale bars are 5 µm. See also Video S7. (H and I) 3D models of PML cages (transparent green) from the same nucleus at higher magnification that reveal the dense packaging of capsids (yellow) and the close association of PML cages with the electron dense heterochromatin (blue). Red spheres represent free capsids. Scale bars are 2 µm. See also Video S8.

These results encouraged us to attempt a 3D reconstruction of the complete volume of a VZV infected cell nucleus in order to visualize and quantify the shape, location, size and number of all PML cages and capsids present. We succeeded in imaging a ribbon of 82 consecutive serial sections (100 nm thickness) through a nucleus (Figure 5A and Video S6). Both the first and last sections contained large areas of heterochromatin, indicating that these sections were cut through the nuclear periphery at the top or bottom of the nucleus, respectively. Therefore, we estimate that an almost complete nuclear volume is represented in this stack of serial sections and in the 3D reconstruction. 3D modeling of the shape of the nucleus from tracing the outer boundary of the nucleus on each section revealed an irregular surface with a wide valley-like indentation (Figure 5B and E). Inspection of the original SSA-SEM images of the serial sections through this nucleus revealed that this wide indentation was directly adjacent to a very prominent ER network in the cytoplasm (Figure 5A, red arrow and Video S6). The total reconstructed nuclear volume was about 291 µm^3^ and contained four distinct PML cages (Figure 5A, black arrows 1–4) of irregularly globular shapes (solid green) (Figure 5C and F) of very different sizes and with volumes that ranged from about 0.8–10 µm^3^ (Table 1). 5,597 nucleocapsids were traced and precisely localized within the reconstructed volume; 5,527 (98.7%) were sequestered within the PML cages (yellow spheres) (Figure 5D and G) and only 70 (1.3%) nucleocapsids (red spheres) were outside of PML cages (Figure 5C–I) (Table 1). Therefore, this comprehensive large volume nuclear reconstruction (Video S7) proved that PML cages are extremely efficient in reorganizing and sequestering thousands of VZV nucleocapsids. Depending on their size, individual PML domains were found to sequester from about 126 capsids to more than 2,700 nucleocapsids with an average packing density of 249±64 SD/µm^3^ (N = 4) (see also Table 1). Again, the four PML cages were found in the periphery of the nucleus associated with the electron dense heterochromatin (Figure 5A, H, I and Video S8).

### PML protein is present in the periphery and the core of PML cages and binds to entrapped VZV capsids

**Figure 6:**
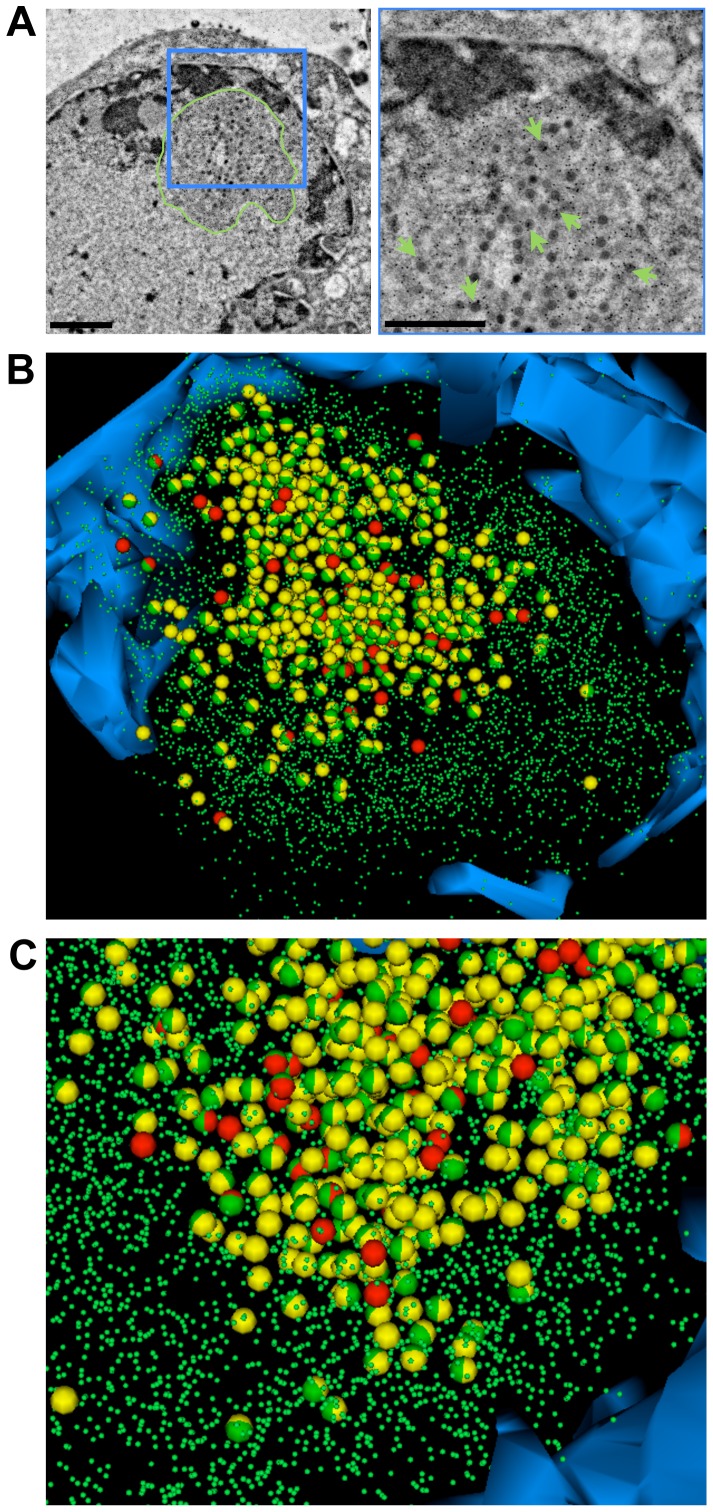
PML protein is associated with entrapped VZV capsids inside PML cages. Melanoma cells that express doxycycline-induced PML IV were infected with VZV for 48 hours and then high pressure frozen, freeze-substituted, embedded in LR-White resin and labeled with anti-PML polyconal rabbit antibody and Protein A conjugated with 15 nm gold particles. (A) A representative TEM image from a series of seven consecutive 100 nm sections is shown. The area in the blue square (left panel) is shown at higher magnification in the right panel. PML specific gold labeling (green arrows) identifies the PML cage (surrounded by a green line, left panel) in the nucleus. Scale bars are 500 nm. (B) The 3D model shows the electron dense heterochromatin (blue) and the location of mature capsids (63; red spheres), immature capsids (403; yellow spheres) and all PML-specific gold particles (5,219; small green spheres) that were identified in the serial sections. Entrapped mature capsids with associated PML labeling are shown as red/green spheres and immature capsids with PML labeling are shown as yellow/green spheres. (C) Same 3D model as in B but at higher magnification and in a different angle. See also Video S9.

The electron density of the PML positive shell of nuclear PML cages allowed tracing and reconstruction of the shape (3D surface view) of this compartment by SSA-SEM; however the 3D distribution of PML protein within PML cages was not revealed using this approach. Therefore we used a serial section immunoTEM (ss-immunoTEM) approach to investigate quantitatively and in three dimensions how PML protein is distributed within the shell and in the core of the PML cages, where the nucleocapsids are entrapped. Seven consecutive sections (100 nm) through HPF/FS-treated and LRwhite embedded cells that contained PML cages with entrapped VZV capsids, were labeled with a PML specific antibody and Protein A conjugated to 15 nm gold particles, and then imaged by TEM (Figure 6A). The results of tracing and modeling of the PML labeling (small green spheres), mature capsids (red spheres) and immature capsids (yellow spheres) and the electron dense heterochromatin (blue) are shown in Figure 6B, C and Video S9. About 5,219 PML gold particles, 63 mature capsids and 403 immature capsids were identified; 272 of the entrapped nucleocapsids were directly associated with PML gold particles (half-green spheres). The 3D reconstruction clearly reveals a ring-shaped ‘cloud’ of dense PML-labeling that corresponds to the electron dense shell of PML cages as seen in the high-contrast embedded samples analyzed by SSA-SEM before. Significant amounts of PML gold labeling were also found in the core of the PML cage (Figure 6A, right panel and Figure 6C) where 58% of the entrapped nucleocapsids were directly associated with PML gold particles (half-green spheres). Therefore PML protein is not only a structural component of the electron dense shell of PML cages but also binds to nucleocapsids entrapped within the core of the PML cages.

### VZV nucleocapsids are entrapped in an electron-dense meshwork in PML cages

**Figure 7:**
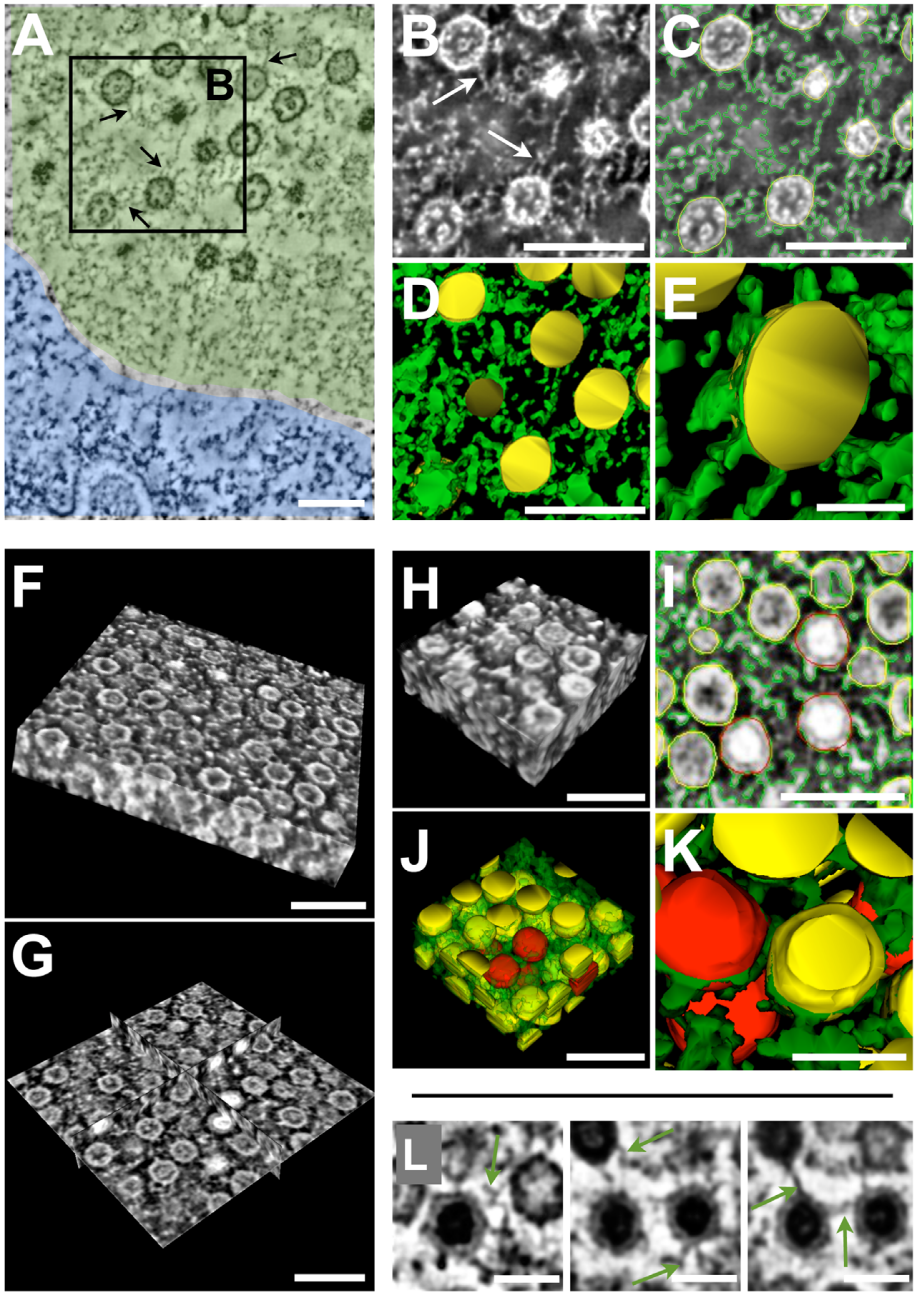
Electron tomography of PML cages reveals the cross-linking of VZV capsids by an electron-dense meshwork. Melanoma cells that express doxycycline-induced PML IV were infected with VZV for 48 h and then high pressure frozen, freeze-substituted and embedded in epoxy-resin. 80 nm sections (A–E) or 300 nm sections (F–L) were investigated by dual-axis electron tomography. (A) A representative tomographic slice shows the periphery of the nucleus with the electron dense heterochromatin (blue bottom area) and part of the PML cage (light green area) containing numerous VZV capsids. A light electron-dense fibrous meshwork (grey) is visible within the PML-domain. These fibers are directly associated with capsids (arrows) and can cross-link them. (B) The area in the black square in A is shown at higher magnification in inverted mode, e.g. electron dense structures appear bright. Arrows depict fibrous material associated with VZV capsids. (C) Same image as in B but with traces for 3D reconstruction shown: capsids (yellow) were traced manually; electron dense meshwork (green) was traced automatically by thresholding. See also Video S10. (D and E) show 3D models of the VZV capsids (yellow) associated with the electron-dense meshwork (green). Scale bars are 200 nm. See also Video S11. (F) Volume view with inverted contrast of a reconstruction from a dual-axis tomogram of a 300 nm section. The arrangement of VZV capsids within a PML cage is visible. (G) Same reconstruction as in F but in orthoslice mode that reveals the arrangement of capsids in the interior of the reconstructed volume. (H) Volume view of a part of the tomographic reconstruction that was then traced and segmented (I) to reveal the position of capsids and the electron dense meshwork in a 3D model. (I) Traces on one representative digital tomographic slice: immature capsids (yellow), mature capsids (red), electron dense fibers and meshwork (green). (J) 3D model shows the packaging of capsids (protein meshwork is green/transparent for unobscured view of capsids). (K) 3D model shows capsids with associated electron-dense meshwork (green) at higher magnification. (L) Representative tomographic slice images that show protein fibers (green arrows) associated with VZV capsids. Scale bars are 200 nm (A–D and F–J) and 100 nm (E, K and L).

The observation by ss-immunoTEM that PML protein was present in the shell and in the center of PML cages, where it was found directly associated with many VZV capsids, suggested that PML protein is not only a structural component of the electron dense shell of PML cages, but may also be involved in the immobilization or cross-linking of sequestered VZV nucleocapsids. To address this hypothesis, we investigated the ultrastructure of PML cages and of sequestered nucleocapsids by electron tomography, which provided a higher resolution than SSA-SEM, albeit at the cost of allowing analysis of only a much smaller (thinner) sample volume. The samples for tomography consisted of HPF/FS-treated and epoxy resin-embedded VZV infected melanoma cells that expressed PML IV together with endogenous PML [22]. We first recorded dual-axis tomograms from 80 nm sections of VZV infected cell nuclei with PML cages (Figure 7A–E). The 3D models were generated by analyzing digital tomogram slices as was done for SSA-SEM, combining manual tracing and automatic threshold-based tracing. EM tomography revealed that all nucleocapsids within PML cages were embedded in an irregular electron dense meshwork with numerous fibrous structures emanating from the nucleocapsids and often cross-linking adjacent capsids (Figure 7A and Video S10). These irregular fibrils were even better visible when the contrast was inverted (Figure 7B, white arrows) and were then traced automatically by applying a threshold (green outline) (Figure 7C) in order to reconstruct a 3D model of the irregular meshwork (green) within PML domains (Figure 7D, E and Video S11). The 3D volume information of tomograms from 80 nm sections is very limited because of the small z-dimension of the section. In order to reveal the precise arrangement and packing of nucleocapsids within the center of the PML cages and to confirm the presence of an irregular electron dense meshwork entrapping VZV nucleocapsids, we next recorded dual-axis tomograms from 300 nm thick sections through PML cages. A volume view representation of a representative tomogram (Figure 7F and Video S12) and an ortho-slice view of the same volume (Figure 7G and Video S12) shows the packing of nucleocapsids in several layers and that, in contrast to paracrystalline inclusion bodies of nucleocapsids observed in some HSV-infected cells [22], those entrapped in PML cages were rather loosely configured, were usually not in direct contact, and the space between them was filled with an irregular electron dense meshwork and fibers. Threshold-aided tracing and 3D reconstructions of the irregular meshwork (green) (Figure 7J–K), and of mature (red) and immature (yellow) VZV nucleocapsids showed that all traced capsids were tightly associated with the irregular meshwork that also cross-linked adjacent capsids (Figure 7I–K and Videos S13 and S14). This cross-linking of adjacent capsids was also visible in the original digital tomogram slices (green arrows) (Figure 7L) and confirmed our observations from the 80 nm tomography reconstructions.

## Discussion

In this work, we developed Serial Section Array-Scanning Electron Microscopy (SSA-SEM), a novel three-dimensional (3D) imaging and reconstruction strategy, and applied the technique to the analysis of VZV-infected cells. Using SSA-SEM and EM tomography, we have reconstructed the nuclei of host cells infected with this representative herpesvirus and, for the first time, revealed the numbers and precise location of thousands of VZV nucleocapsids, visualized the 3D shape and ultrastructure of nuclear PML cages that entrap nucleocapsids, and provided quantitative estimates of the volume, sequestration efficiency and sequestration capacity of these PML cages. The large volume reconstruction of nuclei in VZV-infected cells also provided basic information on how VZV infection affects the shape of the host cell nuclei and how subnuclear domains like electron dense heterochromatin or PML cages and nucleocapsids are spatially related. Of interest, our 3D analysis revealed that PML cages with entrapped capsids were consistently located at the periphery of the nucleus and associated with domains of electron dense heterochromatin, suggesting that the formation of PML cages and VZV capsid sequestration are initiated adjacent to these domains.

Our experimental challenge, which has many similarities to obstacles encountered in addressing other virology and cell biology questions, consisted in how to combine an efficient approach for the large volume 3D reconstruction of infected cell nuclei and complete PML cages with the high ultrastructural resolution necessary to localize VZV nucleocapsids and differentiate mature from immature capsids. Infected cell nuclei have diameters of about 5–10 µm and PML cages are about 0.5–5 µm [22]. These structures are about one order of magnitude too large to be readily reconstructed by conventional electron tomography approaches that usually use 100–300 nm sections. Recent technical and computational improvements have enabled some specialized laboratories to apply serial-sectioning tomography for the reconstruction of large organelles and even complete cells by merging individual tomograms from consecutive sections into a single large volume reconstruction [32]. However, this approach is very labor-intensive so that only a few 3D reconstructions can be generated and this limitation may raise questions about whether these models are fully representative of the structures of interest.

SSA-SEM combines a sample preparation strategy (serial section arrays) similar to the method used in immunofluorescence (IF) array tomography with imaging and detection principles (high resolution SEM with back scattered electron detection) that have been used in serial block face (SBF)-SEM or focus ion beam (FIB)-SEM [27]–[29], [31], [33]. In principle, the latter two methods could also be used to analyze herpesvirus-infected cell nuclei or PML cages. In fact, Feierbach *et al*. used SBF-SEM to locate structures reminiscent of actin filaments and nucleocapsids in cells infected with HSV-1 and PRV, which have caspids that are similar to VZV capsids in size and shape [34]. Bennett *et al*. used FIB-SEM to locate human immunodeficiency virus (HIV) particles in surface-connected tubular conduits in HIV-infected macrophages [30]. However, these approaches require highly specialized equipment that may not be readily accessible. Most importantly, these techniques are destructive imaging methods that destroy the sample block during image stack acquisition by step wise FIB-milling or cutting the sample surface to allow successive surface imaging at different sample levels, while discarding the serially-cut sections. SBF-SEM and FIB-SEM may therefore not be ideal for valuable samples that are difficult to obtain or to prepare. In SSA-SEM, serial sections are secured on a glass slide, creating stable arrays that can be stored and imaged repeatedly, allowing the acquisition of several image series of the same sample at different magnification, resolutions, contrast modes or with different equipment. A major advantage of SSA-SEM is that the interior of cells and tissue become exposed at the section surface, enabling the use of immuno-histochemistry protocols to localize proteins or nuclei acids within the context of the 3D ultrastructure of cells or tissues.

Our 3D reconstructions confirmed that most PML nuclear bodies in VZV infected cells expressing endogenous PML are disassembled efficiently during the course of infection. This process involves the interaction of SUMO-interacting domains (SIM) of the VZV ORF61 protein with sumoylated PML [21]. As a result, most of the several thousand VZV nucleocapsids that were produced in VZV-infected cells appeared randomly distributed in the reconstructed nuclear volume when examined by SSA-SM. As noted, other alphaherpesviruses disrupt PML nuclear bodies and in most cases, also eliminate PML protein by rapid ICP0-mediated degradation [18], [19].

However, when PML disassembly is incomplete, as it is in VZV-infected cells in skin and neural cells *in vivo*, nucleocapsids become sequestered in PML cages. Systematic random sampling analysis of hundreds of ultrathin sections through different PML cages suggested that >95% of all types of VZV nucleocapsids (A, B and C-type) were efficiently sequestered in PML cages [22]. Nevertheless, random ultrathin sections do not reveal the 3D shape and volume of single PML cages because these sections (50–100 nm) may encompass only 1–10% of the diameter of PML cages. Therefore, techniques used in the earlier study did not allow an assessment of the size, volume and shape of PML cages or how many VZV nucleocapsids may be sequestered within individual PML cages. Furthermore, since ultrathin cross-sections through a nucleus encompass only a very small fraction of the nuclear volume, the sequestration efficiency of PML cages could not be determined for single nuclei. These limitations were addressed by using SSA-SEM to reconstruct the shape and volume of individual PML cages, which demonstrated that up to several thousand (2,780) nucleocapsids can be sequestered by single PML cages. Furthermore, quantitative analysis of several thousand nucleocapsids in reconstructed volumes of single nuclei showed that more than 98% of all capsids could become entrapped in PML cages, proving their very high sequestration capacity and explaining the antiviral activity of PML IV [22]. Our method to estimate the sequestration capacity and efficiency of PML cages made it possible to provide information beyond just a morphological description and demonstrates that SSA-SEM can be used in quantitative analyses of virus interactions with nuclear structures.

Given the high sequestration capacity of PML cages, now established by single 3D nuclear analysis and by quantitative random sampling analysis of hundreds of ultrathin cross-sections, it is somewhat surprising that infectious VZV titers were reduced only by about 50% in cell lines expressing PML IV [22]. These results indicate that only very few VZV infectious particles are needed to successfully enter and replicate in adjacent cells. This explanation is consistent with the observation that only very few PRV genomes are required to establish nuclear replication compartments and initiate productive replication, as shown using recombinant PRV, which is also an alphaherpesvirus, carrying a Brainbow cassette [35]. VZV does not release virus particles into the supernatant in cell culture and spreads only from cell to cell by a mechanism that may be facilitated by extensive syncytia formation [5], [7]; therefore, even the few nucleocapsids that may escape sequestration in PML cages should be sufficient to infect adjacent cells *in vitro*. In contrast, in the human host, VZV must infect complex tissues and overcome the barriers of intrinsic and adaptive immunity, which is likely to depend on production of larger numbers of infectious virus particles. Therefore the PML-mediated nuclear sequestration of many VZV capsids observed in human skin or DRG may be expected to have a more substantial antiviral effect [22]. The quantitative analysis of the different types of capsids present within infected cell nuclei revealed that the majority (70–90%) were immature (A and B-type capsids) while only a minority was in a mature stage (C-type, 10–30%). We speculate that large numbers of immature nucleocapsids help to outcompete the limited sequestration capacity of PML cages, giving mature capsids a better chance to egress from the nucleus. These observations also suggest that the relatively few mature virions observed in VZV infected cells *in vitro* is not just a tissue culture phenomenon.

Using conventional EM tomography, we obtained the first insights about the 3D ultrastructure of PML cages, suggesting how VZV nucleocapsids may be kept entrapped in these nuclear domains. Tomographic 3D reconstructions revealed the presence of an electron dense meshwork surrounding sequestered nucleocapsids and fiber-like like structures, that often cross-linked adjacent nucleocapsids, suggesting that capsids were entrapped by restricting their mobility and ‘gluing’ them together. The 3D analysis of PML-labeled sections by serial section immunoTEM showed that PML protein was present both in the periphery of the cage (the ‘shell’) and associated with the capsids entrapped in the center of PML cages. PML protein which is the main structural component of PML nuclear bodies, forms homo-and heterooligomers [10]; therefore at least part of the electron dense meshwork is likely to consist of PML-oligomers that crosslink and embed capsids in a protein meshwork. The PML-positive meshwork and fibers were in general directly associated with the edges of VZV capsids, which is consistent with our previous biochemical data that demonstrated an interaction of PML with the small outer capsid protein ORF23 [22]. Many other proteins resident in PML-nuclear bodies, e.g. hDaxx or Sp100, may be part of this meshwork [9].

Cryo-tomography is an alternative that would enable a 3D reconstruction of PML cages at even higher resolution and more native conditions (avoiding resin embedding and heavy metal staining) but this approach can be predicted to encounter major experimental challenges. Frozen-hydrated sections through cell nuclei will be required, the identification of PML cages will be very demanding as these structures are not abundant, and identification would need to occur at a very low electron dose that creates noisy imaging conditions. Molecular docking of known crystal structures to electron densities will also be difficult, because of the many other proteins present in PML nuclear domain and currently only the PML RING domain has been crystallized [36], whereas the PML IV C-terminal domain is critical for nucleocapsid sequestration [22].

In summary, we were able to create complete reconstructions of herpesvirus-infected cell nuclei and PML nuclear domains in three dimensions for the first time using 3D SSA-SEM and EM tomography. This study supports and extends our recent discovery and characterization of PML cages that efficiently sequester VZV nucleocapsids in cell culture and in differentiated human skin and neural cells infected *in vivo* and represents a novel antiviral mechanism, distinct from the established role of PML in controlling several alphaherpesviruses shortly after virus entry by limiting early viral gene transcription. Visualization of the shape and measurements of the volumes of host cell nuclei and PML cages together with the 3D localization of VZV nucleocapsids with ultrastructural precision enabled us to determine the sequestration efficiency and capacity of PML nuclear cages. This work contributes not only to a more comprehensive understanding of the antiviral activity of PML cages against VZV, a pathogenic human herpesvirus, but also provides a novel method to undertake the 3D reconstruction and quantitative investigation of nuclear PML domains that have also been found to be associated with capsids of papillomaviruses and polyomaviruses [37]–[39]. The method has broad relevance for addressing other questions in virology and cell biology where large volume 3D reconstruction with high precision imaging of intracellular structures is needed.

## Materials and Methods

### Cells and viruses

The human melanoma cell line (MeWo, ATCC number: HTB-65) was grown in Dulbecco's modified Eagle's medium supplemented with 10% fetal bovine serum, nonessential amino acids (100 µM) and antibiotics (penicillin at 100 U/ml and streptomycin at 100 µg/ml). Melanoma cells were passaged fewer than 25 times. Melanoma cells expressing doxycyline-inducible PML IV were constructed using the pRetro-X-Tet-On-Advanced vector system and pRetro-X-Tight-Pur plasmid (Clontech Laboratories) with the PML IV plasmid pcDNA3-PML IV as described recently [22]. The stable cells were induced with 5 µg/ml doxycyline for 24 hr before infection with VZV. The virus was recombinant Oka (rOka) derived from the wild type low passage parent Oka strain (pOka). Viral infection was done with cell-associated VZV at a ratio of 1/20 (infected cells/uninfected cells) for 48 hr.

### Antibodies and Confocal Immunofluorescence Microscopy (IF)

Cultured cells on glass coverslips were fixed in 4% paraformaldehyde in PBS for 20 min at room temperature. Cells were blocked and immunostained as described previously [5]. Antibodies used for confocal microscopy were: mouse monoclonal anti-PML (PG-M3) from Santa Cruz Biotech and rabbit polyclonal anti-VZV-ORF23 described previously [5], [22]. Secondary antibodies were Alexa Fluor 488 and Alexa Fluor 594 conjugated donkey anti-mouse or donkey anti-rabbit antibodies (Invitrogen). Infected cultured cells were imaged using a Leica TCS^SP2^ confocal laser scanning microscope (Heidelberg, Germany). Microscope objectives were 40×/1.0 (Numerical Aperture, N.A.) or a 63×/1.4 (N.A.) Plan Apochromat objectives. Images were scanned at 1024×1024 pixels with at least four times frame averaging and the pinhole adjusted to one airy unit. Brightness and contrast were adjusted using Photoshop CS3 (Adobe) or iPhoto (Apple).

### Sample preparation for Transmission Electron Microscopy (TEM), immuno-TEM and tomography

For standard TEM, samples were fixed in 4% paraformaldehyde and 2% glutaraldehyde in 0.1 M phosphate buffer (ph 7.2) and embedded in epoxy-resin. For immuno-TEM or EM tomography samples were fixed in 4% paraformaldehyde and 0.1% glutaraldehyde in 0.1 M phosphate buffer (ph 7.2) and then high-pressure frozen (HPF) in a Leica EM PACT2 and freeze substituted (FS) in either LR-white or Epoxy resin (Embed812), respectively. Frozen specimen carriers with cells were placed into frozen cryovials containing acetone with 0.1% glutaraldehyde and 0.1% uranyl acetate (for LR White embedding) or in acetone with 1% osmium tetroxide and 0.1% uranyl acetate (for Epon embedding). The frozen vials were then placed into a Leica AFS for the freeze-substitution procedure and then embedded in either or LR-White resin for immuno-TEM or epoxy resin Embed 812 for EM tomography. Sections (80–300 nm) were prepared with a diamond knife (Diatome) using an ultramicrotome (Ultracut, Leica). For immunogold-labeling LRwhite sections were pre-blocked in DIG-blocking solution (Roche) for 30 min. Primary antibodies and Protein A-gold particles (obtained from CMC, Utrecht, the Netherlands) were diluted in blocking solution and sections were incubated for 1 h or 30 min, respectively, at RT. Rabbit polyclonal anti-PML antibody (Santa Cruz Biotech) was used at 1∶10 dilution. Sections were stained with 3.5% aqueous uranyl acetate for 15 minutes and with 0.2% lead citrate for one minute and air-dried. Sections were analyzed using a JEOL 1230 transmission electron microscope (TEM) at 80 kV and digital photographs were captured with a GATAN Multiscan 701 digital camera.

### Serial section array scanning electron microscopy (SSA-SEM)

VZV-infected cells were fixed in 4% paraformaldehyde/2% glutaraldehyde in 0.1 M phosphate buffer (pH 7.2) for 24 hr and cell pellets were stabilized by embedding in 10% gelatine. The samples were washed with ultrapure water and then postfixed with 2% osmium tetroxide reduced with 1.5% (w/v) potassium ferrocyanide for 2 hr at room temperature. The samples were then washed again, followed by one hour incubation with 1% (w/v) tannic acid, washing in ultrapure water and a final “en block” staining with 3.5% (w/v) uranyl acetate over night. The samples were then dehydrated in a series of ascending ethanol concentrations (30%–100%), treated with propylene oxide and finally embedded in epoxy resin Embed 812 (Electron Microscopy Sciences, Inc.). The procedure for the preparation of Serial Section Arrays (SSA) was similar to the method described for fluorescence array tomography [27], [33], [40]: serial sections (100 nm thickness) were cut with a jumbo histo diamond knife (Diatome) and collected onto precleaned glass slides that had been coated with a solution of 0.3% gelatine with 0.1 g/l chromium potassium sulphate. To enable SEM imaging, the SSAs were finally counter stained with uranyl acetate and lead citrate and then heavily carbon coated (two cycles of 30 seconds until the surface color was light brown) using a Benchtop Turbo III apparatus (Denton Vacuum, LLC) and attached to SEM stubs using colloidal graphite or adhesive copper tape (both from EMS, Inc).

The arrays were first pre-scanned with a Hitachi S-3400N VP-SEM to assess the quality of the ribbons of serial sections and to find regions of interest (ROIs). ROIs were mapped at a magnification from 100× (whole section image) to 10,000× (image of cell nucleus with resolved capsids) using an accelerating voltage of 10 kV, a working distance of 8.5 mm and the back-scattered electron (BSE) detector. For the final acquisition of high-resolution digital image stacks from serial sections, the arrays were transferred to a Zeiss Sigma FE-SEM that is equipped with a field emission gun (FE). The mapped ROIs were identified and then imaged using the BSE detector at magnifications from 5,000–40,000× with an accelerating voltage from 6 kV–10 kV and a working distance from 6–7 mm; images were scanned at 2048×1536 pixels, with at least 2 times line averaging.

### 3D visualization of SSA-SEM image stacks

SSA-SEM image stacks were automatically aligned (registration) in rigid mode using the ‘StackReg’ plugin in the Fiji/ImageJ software package (http://fiji.sc/wiki/index.php/Fiji). Aligned images were saved as image sequence files and then imported into the 3D reconstruction program ‘Reconstruct’(http://synapses.clm.utexas.edu/tools/reconstruct/reconstruct.stm) [41]. Segmentation of cells and infected nuclei was accomplished by manual classification and tracing the boundary contours of ultrastructures of interest. The visualization of the 3D shape of cell nuclei (grey), heterochromatin (blue), protein aggregates (brown) and PML domains (green) was achieved by representing the traces of these objects as Boissonnat surfaces using “Reconstruct” [41]. Cross-sectioned nucleocapsids, which are rotation-symmetric icosahedral structures, were traced and modeled as 3D spheres with 100 nm diameters. A section thickness of 100 nm was deliberately chosen to avoid double counting of the same capsids (which have a diameter of about 100 nm) in consecutive sections. Because of the anisotropic resolution in the serial section reconstructions the position of VZV nucleocapsids is more precisely modeled in the x–y dimension than in the z-dimension (100–200 nm resolution). Volumes of reconstructed nuclei and of PML cages were calculated and the number of VZV capsids and PML gold particles were counted using the corresponding traces in the ‘Reconstruct” software. The 3D models were saved as 360° image series and then exported into Fiji/ImageJ, where the files were compressed (JPEG) and saved as movie files (.avi).

### TEM tomography

VZV infected cells were fixed and high pressure frozen (HPF), freeze substituted (FS) and embedded in epoxy resin (Embed812) as described above (TEM sample preparation). 80 nm or 300 nm sections were cut with a diamond knife (Diatome) using an ultramicrotome (Ultracut, Leica) and placed on Formvar and carbon coated 75mesh TEM copper grids (TedPella). Sections were stained with 3.5% aqueous uranyl acetate for 15 minutes and with 0.2% lead citrate for one minute and air-dried. Finally, the sections were coated on both sides of the grid with 15 nm colloidal gold particles (Ted Pella) as fiducial markers by repeated dipping of the grids in the colloidal gold solution followed by air drying. The 80 nm thick sections were imaged on a JEOL 1400 TEM (JEOL USA, Inc.) at 120 kV equipped with a dual-axis tomography holder. The double-tilt series were recorded with the ‘SerialEM’ software package (http://bio3d.colorado.edu/SerialEM/) using a tilt range of ±65° at 1.5° angular increments [42]. The image pixel size ranged from 0.43–1.3 nm. The 300 nm thick sections were imaged on a Titan ETEM (FEI company, USA) operated at 300 kV using a dual-axis tomography holder. Double tilt series were recorded with a tilt range of ±65° at 1.5° angular increments using the Xplore3D software (FEI). Image pixel size was 1.4 nm at the specimen level.

The series of dual-axis tilt images were aligned, reconstructed by weighted back-projection and then merged into dual-axis tomograms using the software package IMOD 4.1 (http://bio3d.colorado.edu/imod/) [43], [44]. The stack of digital tomogram slices (.rec file) was imported into Fiji/ImageJ and saved as image sequence file that was then imported into the ‘Reconstruct’ software for tomogram segmentation and 3D modeling. VZV capsids were traced manually and visualized using Boissonnat surfaces. The electron dense meshwork within the PML cages was traced automatically applying a threshold and the ‘wild fire’ tool in the ‘Reconstruct’ software; 3D visualization of the meshwork was also achieved using Boissonnat surfaces [41]. Volume views and ortho-slice views of the tomograms were generated by importing the digital tomogram slices (.rec file) into Fiji/ImageJ and applying the ‘volume viewer’ plug-in.

### Statistical analysis

Graph Pad Prism (version 5.0) statistical software was used for quantification and statistical analysis.

## Supporting Information

Video S1 Corresponds to Figure 3A and shows an animation through a z-series of 100 nm thick serial sections imaged by BSE-SEM illustrating the interior of the nucleus of a VZV infected melanoma cell. VZV capsids are visible as 100 nm particles.
(AVI)

Video S2 Corresponds to Figure 3B–F and shows animations of the segmented volume of the nucleus shown in Figure 3A and in Video S1. The sequence of the animations in the video corresponds to the views seen in Figure 3B, C, D and E, in this order. The 3D models show the outer boundary of the nucleus (grey), the electron dense heterochromatin (blue), the nucleolus (brown) and the location of all mature capsids (red spheres) and immature capsids (yellow spheres).
(AVI)

Video S3 Corresponds to Figure 3G and shows the animation of the segmented volume of the nucleus of a VZV infected melanoma cell that was reconstructed from a stack of serial sections (100 nm thick) imaged by TEM. The 3D model shows the electron dense heterochromatin (blue), the nucleolus (brown) and the location of all mature capsids (red spheres) and immature capsids (yellow spheres).
(AVI)

Video S4 Corresponds to Figure 4B and shows an animation of an image stack obtained by SSA-SEM illustrating the interior of the nucleus of a VZV infected melanoma cell that contains numerous VZV capsids sequestered in two electron dense PML cages. VZV capsids are visible as 100 nm particles.
(AVI)

Video S5 Corresponds to Figure 4C and D and shows animations of the segmented volume of the nucleus shown in Figure 4B and in Video S4. The sequence of the animations corresponds to the views seen in Figure 4C and D. The 3D models show the electron dense heterochromatin (blue), protein aggregates (brown), VZV capsids (yellow spheres) and two PML cages (green). The last animation in the sequence also reveals the boundary of the reconstructed volume (grey).
(AVI)

Video S6 Corresponds to Figure 5A and shows an animation through an image stack obtained by SSA-SEM, illustrating the interior of the nucleus of a VZV infected melanoma cell that contains >5,500 VZV capsids sequestered in four spherical PML cages. VZV capsids are visible as 100 nm particles.
(AVI)

Video S7 Corresponds to Figure 5B–G and shows animations of the segmented volume of the nucleus shown in Figure 5A and in Video S6. The sequence of the animations corresponds to the views seen in Figures 5B and E (shape of the nucleus, grey), Figures 5C and F (heterochromatin, transparent blue; PML cages, solid green and unsequestered VZV capsids, solid red) and Figures 5D and G (heterochromatin, transparent blue; unsequestered VZV capsids, solid red and sequestered capsids, solid yellow). To reveal the sequestered VZV capsids, the PML cages are shown completely transparent in the last animation.
(AVI)

Video S8 Corresponds to Figure 5H and I and shows animations of the segmented volume of the nucleus shown in Figure 5A and in Video S6. It shows the close association of PML cages (transparent green to reveal the sequestered capsids) with the dense heterochromatin (solid blue) in the periphery of the nucleus. Unsequestered VZV capsids are visible as solid red spheres.
(AVI)

Video S9 Corresponds to Figure 6B and C and shows animations of the segmented volume of a PML cage with sequestered VZV capsids that was reconstructed from seven serial 100 nm immuno-TEM sections. The sequence of the animations corresponds to the views seen in Figures 6B and C, and shows heterochromatin (blue), PML gold labeling (green particles), mature capsids (red spheres) and immature capsids (yellow spheres). VZV capsids with associated PML gold labeling are shown as half-green spheres.
(AVI)

Video S10 Corresponds to Figure 7A and shows a stack of digital slices obtained from the tomographic reconstruction of a 80 nm section through a host cell nucleus with VZV capsids sequestered in a PML cage. The video shows the digital slices first in normal mode (electron dense structures appear dark) and then in inverted mode (electron dense structures appear bright, for better visibility of the fibers and the meshwork that are associated with the VZV capsids.)
(AVI)

Video S11 Corresponds to Figure 7D and E and shows animations of segmented areas of the tomographic slices shown Figure 7A–C. The animations reveal the close association and cross-linking of VZV capsids (yellow spheres) with electron dense material (green) within PML cages.
(AVI)

Video S12 Corresponds to Figure 7F and G and shows animations of a tomographic reconstruction of a 300 nm section through a PML cage with sequestered VZV capsids. The video shows first the animated volume view of the tomogram revealing the arrangement and packaging of capsids, followed by an animated ortho-slice view (revealing views of cross-sections through the middle of the reconstructed volume). Then the stack of digital slices of the tomogram is shown in inverted mode (electron dense structures appear bright) and finally the same stack is shown in normal mode (electron dense structures appear dark).
(AVI)

Video S13 Corresponds to Figure 7J and shows animations of the segmented tomographic volume shown in Figure 7H (which is part of the tomogram shown in Figure 7F). The animations reveal the arrangement and packaging of mature (red) and immature (yellow) capsids in the reconstructed volume. The electron dense meshwork of the PML domain is shown in transparent green.
(AVI)

Video S14 Corresponds to Figure 7K and shows animations of the segmented tomographic volume shown in Figure 7H. The animations reveal the arrangement of mature (red) and immature (yellow) capsids and show their association and cross-linking with an electron dense meshwork (solid green) in the reconstructed volume of a PML cage.
(AVI)
